# One Health Surveillance for SARS-CoV-2 in Non-Human Primates and Small Mammals in Minas Gerais, Brazil

**DOI:** 10.3390/pathogens14040356

**Published:** 2025-04-06

**Authors:** Pedro Augusto Almeida-Souza, Thamires Gabriele Macedo Silva, Gabriele Barbosa Penha, Thaynara de Jesus Teixeira, Ramon Oliveira-Silva, Iago Alves Celestino, Maria Eduarda Gonçalves-dos-Santos, Cirilo Henrique de Oliveira, Alice dos Santos Nunes Ferreira, Emerson Márcio Gusmão, Vinícius de Oliveira Ottone, Danilo Simonini-Teixeira, Fabrício Souza Campos, Paulo Michel Roehe, Leonardo Camilo de Oliveira, Mauro Martins Teixeira, Filipe Vieira Santos de Abreu, Danilo Bretas de Oliveira

**Affiliations:** 1 Laboratório de Comportamento de Insetos, Instituto Federal do Norte de Minas Gerais, Salinas 39560-000, MG, Brazil; pedro.aasouza2020@gmail.com (P.A.A.-S.); gbpenha1@gmail.com (G.B.P.); thaynarateixeira701@gmail.com (T.d.J.T.); ramonos.oliveira@gmail.com (R.O.-S.); iagoalves958@gmail.com (I.A.C.); dudasantos31082016@gmail.com (M.E.G.-d.-S.); cirilohenrique15@gmail.com (C.H.d.O.); 2 Laboratório de Inovação Tecnológica e Empreendedorismo em Controle de Vetores, Departamento de Parasitologia, Universidade Federal de Minas Gerais, Belo Horizonte 31270-901, MG, Brazil; 3 Laboratório de Doenças Parasitárias, Universidade Federal do Vale do Jequitinhonha e Mucuri, Diamantina 21040-900, MG, Brazil; thamires.macedo@ufvjm.edu.br (T.G.M.S.); alice.nunes@ufvjm.edu.br (A.d.S.N.F.); emersonmarciob6@gmail.com (E.M.G.); vinicius.ottone@ufvjm.edu.br (V.d.O.O.); 4 Núcleo de Atendimento e Pesquisa de Animais Silvestres, Departamento de Ciências Agrárias e Ambientais, Universidade Estadual de Santa Cruz, Ilhéus 45662-900, BA, Brazil; dsteixeira@uesc.br; 5 Instituto de Ciências Básicas da Saúde, Universidade Federal do Rio Grande do Sul, Porto Alegre 90035-003, RS, Brazil; camposvet@gmail.com (F.S.C.); proehe@gmail.com (P.M.R.); 6 Departamento de Bioquímica e Imunologia, Instituto de Ciências Biológicas, Universidade Federal de Minas Gerais, Belo Horizonte 31270-901, MG, Brazil; ldeolive2@gmail.com (L.C.d.O.); mmtex.ufmg@gmail.com (M.M.T.); 7 Laboratório de Insetos Transmissores de Hematozoários, Instituto Oswaldo Cruz, Fundação Oswaldo Cruz, Rio de Janeiro 21040-900, RJ, Brazil

**Keywords:** COVID-19, Rodentia, Marsupialia, Platyrrhini, molecular surveillance, one health

## Abstract

Although the SARS-CoV-2 pandemic primarily affected the human population, the virus has also been detected in various animal species worldwide, raising concerns about its potential to establish new animal reservoirs. This study aimed to investigate the presence of SARS-CoV-2 in non-human primates (NHPs) and synanthropic small mammals (SSMs) in the Jequitinhonha Valley and Northern Minas Gerais, Brazil. Between October 2021 and October 2023, 119 animals were sampled, 82 NHPs and 37 SSMs, across 22 municipalities. A total of 342 biological samples—including oral and nasal swabs, lungs, livers, spleens, blood, and feces—were collected and analyzed using RT-qPCR, while 37 serum samples were submitted to neutralization tests. Despite the diversity of sampled species, habitats, and biological materials, no evidence of SARS-CoV-2 infection or specific antibodies was detected in any of the individuals tested. The results suggest that NHPs and SSMs in these regions did not act as reservoirs for SARS-CoV-2 during the study period. This finding is particularly relevant given the high synanthropy of species such as *Callithrix penicillata* (black-tufted marmoset) and *Rattus rattus* (black rat), which frequently interact with human populations. Our study underscores the importance of integrating animal, human, and environmental health perspectives under a One Health framework to monitor emerging zoonotic threats. By providing baseline data on SARS-CoV-2 dynamics in wildlife, we emphasize the need for ongoing ecological and epidemiological surveillance to assess potential spillover events and their implications for biodiversity and public health in Brazil.

## 1. Introduction

Severe acute respiratory syndrome coronavirus 2 (SARS-CoV-2), a species named *Betacoronavirus pandemicum*, belonging to the family *Coronaviridae* and the genus *Betacoronavirus*, is the causative agent of coronavirus disease 2019 (COVID-19) [1]. First detected in Wuhan, China, in December 2019, SARS-CoV-2 led to a pandemic, officially declared by the World Health Organization (WHO) on March 11, 2020. In Brazil, the first confirmed case of COVID-19 was reported on February 26, 2020, in the state of São Paulo [2], with the disease quickly spreading nationwide.

On 5 May 2023, the World Health Organization (WHO) declared the end of the Public Health Emergency of International Concern for COVID-19 [3]. Since the onset of the pandemic, approximately 770 million cases and nearly 7 million confirmed deaths have been reported worldwide [4]. However, the impact of SARS-CoV-2 extends beyond human infections, affecting various animal species globally, including domestic animals [5,6], livestock [7], and captive animals housed in zoos and wildlife rescue centers [8]. Additionally, non-human primates (NHPs), synanthropic rodents, and marsupials—often considered urban pests—have been identified as potential hosts for the virus [9,10].

The emergence of new animal species capable of hosting SARS-CoV-2 raises growing concerns about its potential impact on the survival of threatened species and its ability to alter the virus’s transmission dynamics. Studies suggest that species such as minks and white-tailed deer may have already become wildlife reservoirs for the virus, increasing the risk of secondary spillover events to humans [11,12,13]. These spillover events are particularly concerning as they could introduce new mutations into human populations. From a One Health perspective, such events highlight the urgent need for continuous surveillance to assess the risk of SARS-CoV-2 becoming an anthropozoonosis—a disease capable of circulating between humans and animals [14,15,16,17,18].

In this context, NHPs are particularly important due to their genetic, anatomic, and physiological proximity to humans. Old World primates exhibit high susceptibility to the virus, as their angiotensin-converting enzyme 2 (ACE2) is structurally similar to that of humans, facilitating viral entry into host cells and making these animals more vulnerable to infection [19,20]. In Brazil, NHPs such as the genus *Callithrix* (marmosets) often display synanthropic behaviors and maintain close contact with humans, increasing the risk of infection as documented in other wild animals in the United States [21]. Moreover, SARS-CoV-2 infection in NHPs has been reported to be potentially fatal [22,23], raising concerns about the conservation of these species.

In contrast to NHPs, small synanthropic mammals (SSMs), such as rodents and marsupials, may serve as urban reservoirs for SARS-CoV-2 despite having lower structural similarity in their ACE2 receptors [19]. These animals thrive in high densities in urban areas and frequently interact with humans in search of food waste or sewage, increasing their potential role in viral maintenance and transmission [24,25,26]. Consequently, monitoring these small mammals is essential for tracking viral circulation and mitigating the risk of potential zoonotic outbreaks.

Therefore, this study aimed to assess the SARS-CoV-2 infection rate in non-human primates (NHPs) and synanthropic small mammals (SSMs) in the Jequitinhonha Valley and Northern Minas Gerais regions of Brazil. Despite their rich biodiversity, these areas face significant economic challenges, which increase the vulnerability of the human population and local wildlife to zoonotic outbreaks and other public health risks. This study is part of a broader initiative within the Yellow Fever Surveillance Project in Brazil (Febre Amarela BR—project no. 443215/2019-7) and the Wastewater-Based Epidemiology Network for COVID-19 Monitoring (REMONAR—project no. 400284/2022-7). Both projects seek to integrate epidemiological data from multiple sources, monitoring viral circulation in humans, animals, and the environment. This interconnected approach is crucial for implementing a “One Health” framework, which recognizes the interdependence of human, animal, and environmental health.

## 2. Materials and Methods

### 2.1. Capture Efforts and Sampling Methods

Between October 2021 and October 2023, NHPs and SSMs were captured using Tomahawk traps baited with various food combinations. For NHPs, the traps contained banana and mango, while for SSMs, a mix of bacon, banana, bread, and honey was used [27,28].

**Figure 1:**
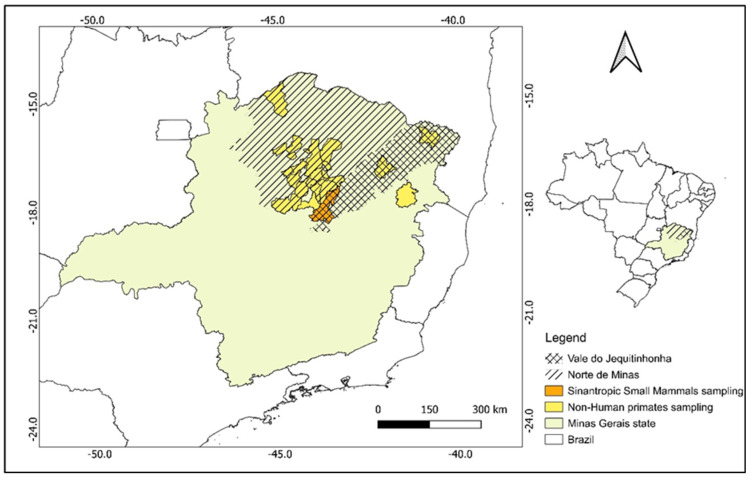
Regions and municipalities where animals were sampled.

NHP sampling was conducted in urban, rural, and wild areas across 20 municipalities in the Jequitinhonha Valley and Northern Minas Gerais, regions predominantly covered by Cerrado vegetation (Figure 1). Urban landscapes were defined as areas with high human density and infrastructure, including residential, commercial, or industrial zones. Rural areas encompassed agricultural lands and sparsely populated regions with fragmented natural vegetation. Wild environments comprised preserved or minimally disturbed natural habitats with limited human influence. Captured monkeys were anesthetized with a combination of ketamine hydrochloride (15 mg/kg) and midazolam (0.5 mg/kg), following the Brazilian Ministry of Health protocol [28]. Following anesthesia, oral swab samples were collected. Additionally, as part of the yellow fever surveillance project, all NHPs found dead during the capture period were examined, and lung samples were collected.

On the other hand, SSMs were captured exclusively within the urban perimeter of Diamantina between June and September 2021. Traps were placed in areas with vegetation cover and natural watercourses contaminated with domestic sewage—locations where SARS-CoV-2 was later confirmed [29]. Captured SSMs were euthanized using a combination of ketamine (90 mg/kg) and xylazine (5 mg/kg). Blood, feces, and body and nasal swab samples were collected for SARS-CoV-2 detection.

All samples from NHPs and SSMs were immediately preserved in RNAlater solution (Thermo Fisher) and liquid nitrogen in the field before being transported to the laboratory, where they were stored at −80 °C until processing.

All methods and protocols were approved by the Institutional Ethics Committee for Animal Experimentation (Protocol CEUA/IFNMG no. 14/2019 and CEUA/UFVJM no. 002/2021) and authorized by the Brazilian Ministry of the Environment (SISBIO no.71,714-2 and no. 75,445-1).

### 2.2. RNA Extraction and Molecular Detection by RT-qPCR

RNA extraction from NHP samples was performed using the PureLink RNA Mini Kit (Invitrogen, Thermo Fisher Scientific—Waltham, MA, USA) or the RNeasy Mini Kit (Qiagen, Hilden, Germany), following the manufacturer’s protocols. The detection protocols for SARS-CoV-2 in NHPs and SSMs differed because the analyses were performed in different laboratories, each applying standardized protocols optimized for the available infrastructure. Molecular detection of SARS-CoV-2 genetic material in all NHP samples was conducted using the SARS-CoV-2 Molecular Kit—EDx (Bio-Manguinhos, Rio de Janeiro, Brazil). Briefly, the primers used targeted the envelope gene (E_Sarbeco_F1: ACAGGTACGTTAATAGTTAATAGCGT; E_Sarbeco_R2: ATATTGCAGCAGTACGCACACA; and E_Sarbeco_P1: FAM-ACACTAGCCATCCTTACTGCGCTTCG-NFQ), with an extraction control for the RNAse-P gene (RdRP_SARSr-F2: GTGARATGGTCATGTGTGGCGG; RdRP_SARSr-R1: CARATGTTAAASACACTATTAGCATA; and RdRP_SARSr-P2: VIC-CAGGTGGAACCTCATCAGGAGATGC-NFQ) [30]. The cycling conditions were as follows: 50 °C for 15 min, 95 °C for 2 min, followed by 40 cycles of 95 °C for 15 s and 58 °C for 30 s. The kit also included a positive control. Samples were considered negative if the cycle threshold (Ct) was above 40, provided that the extraction and positive controls showed a Ct below 40, indicating successful reaction and nucleic acid extraction.

For SSM samples, viral RNA from nasal and body swabs was extracted using the Invitrogen Thermo Fisher Scientific RNA Kit, while RNA from fecal samples was extracted using the Trizol (Invitrogen, Thermo Fisher Scientific, Waltham, MA, USA) method, following the manufacturers’ instructions for both protocols. SARS-CoV-2 RNA was detected using the Allplex™ 2019-nCoV Assay (Seegene, Seoul, South Korea), which enables the simultaneous amplification of multiple genomic targets, including the E (envelope), S (spike), RdRP (RNA-dependent RNA polymerase), and N (nucleocapsid) genes (primer sequences not provided by the manufacturer). RT-qPCR assays were performed according to the manufacturer’s instructions. The kit’s positive control was used to validate reaction efficiency and confirm the absence of inhibitors. Amplification conditions were as follows: reverse transcription at 50 °C for 20 min, initial denaturation at 95 °C for 15 min, followed by 45 amplification cycles (94 °C for 15 s; 58 °C for 30 s). Samples were considered negative if the Ct for all targets was above 40.

### 2.3. Serological Assay-Plaque Reduction Neutralization Test (PRNT) Anti-SARS-CoV-2

Serum samples from SSMs were heat-inactivated at 56 °C for 20 min and tested in duplicate using PRNT, as previously described [31]. Briefly, 1 × 10^5^ Vero CCL-81 cells per well were seeded into 24-well plates 24 h before infection with SARS-CoV-2. The viral suspension was serially diluted in DMEM-2 to obtain 200 plaque-forming units (PFUs) in 100 µL (final concentration: 2 × 10^3^ PFU/mL). Each serum sample was serially two-fold diluted, starting at 1:20 in 100 µL of DMEM-2, up to 1:80. For each serum dilution, 100 μL of the viral suspension containing 200 PFU was added (final volume = 200 µL). A virus control sample was incubated with a non-reactive human serum as a reference. The mixture was agitated at 200 rpm at 37 °C for 1 h, after which 100 µL was inoculated onto Vero cells. The infected plates were incubated at 37 °C for 1 h and then overlaid with 1 mL per well of DMEM-1% fetal calf serum (FCS) supplemented with 1.2% Carboxymethylcellulose (CMC) overlay medium. Plates were further incubated at 37 °C for 72 h until individual viral lysis plaques appeared. Following incubation, infected cells were fixed with 10% buffered formaldehyde solution for 15 min and stained with 1% crystal violet solution for 15 min. For each test, anti-SARS-CoV-2 horse serum was used as a PRNT-positive control. The neutralizing activity of the serum against SARS-CoV-2 was determined and expressed as the serum dilution required to achieve a 50% reduction in PFUs compared to the virus control.

## 3. Results

A total of 119 individuals were captured, and 342 samples were tested by RT-qPCR. This included 82 NHPs, from which we analyzed 83 samples (32 oral swabs and 51 liver samples), and 37 SSMs, from which 259 samples were analyzed. The SSM samples comprised oral/nasal swabs, lung, body swabs, blood, liver, spleen, and feces from each specimen. The sampled individuals represented eight different species: *Alouatta caraya* (black-and-gold howler monkey), *Callithrix geoffroyi* (white-headed marmoset), *C. khulii* (Wied’s marmoset), *C. penicillata* (NHPs) and *Didelphis albiventris* (white-eared opossum), *Mus musculus* (house mouse), *Rattus norvegicus* (brown rat), and *Rattus rattus* (SSMs). These individuals were distributed across 22 municipalities during and after the SARS-CoV-2 pandemic (Table 1). Among the NHPs, *C. penicillata* (black-tufted marmoset) was the most frequently sampled species, accounting for 71 individuals (83.5%), likely due to its widespread regional distribution and synanthropic behavior. *R. rattus* was the most commonly captured species among the SSMs, with 18 individuals (48.6%).

Most animals were collected in urban landscapes (77 individuals, 64.7%), followed by rural areas (36 individuals, 30.2%) and wild environments (6 individuals, 5%). A total of 307 biological samples were analyzed, including oral and body swabs, blood, serum, lungs, liver, and spleen (Table 1).

Despite the broad sampling, which included species with varying degrees of synanthropy, diverse capture locations, and multiple sample types, no evidence of SARS-CoV-2 RNA was detected in any of the analyzed individuals (Table 1). Additionally, all 37 SSM serum samples tested for SARS-CoV-2-specific antibodies using the PRNT assay were negative.

## 4. Discussion

The COVID-19 pandemic posed significant risks to human health, underscoring the intricate connections between humans, animals, and the environment. In this context, the potential spillover and spillback of SARS-CoV-2 into wildlife raised global concerns, mainly due to the risk of establishing zoonotic cycles with ecological and public health implications. By investigating the presence of SARS-CoV-2 in NHPs and SSMs from the Jequitinhonha Valley and Northern Minas Gerais, this study provides evidence supporting that these species are free from SARS-CoV-2 infection while also promoting broader discussions on wildlife–pathogen interactions and their implications for emerging infectious diseases.

Non-human primates typically live in social groups, and some species—particularly Platyrrhini primates of the genus *Callithrix*—exhibit synanthropic behavior, frequently occurring in rural and urban environments or in ecotopes, especially during dawn and dusk. In this study, 76 out of 82 captured NHPs originated from rural or urban areas, environments that increase the likelihood of human interaction and, consequently, potential exposure to SARS-CoV-2 [21]. Despite these factors, none of the collected samples resulted positive for SARS-CoV-2 RNA.

Similar findings have been reported in other studies conducted in Brazil. A total of 60 free-ranging urban NHPs from Manaus and São José do Rio Preto—cities with high SARS-CoV-2 prevalence during the sampling period—were screened, but none tested positive for the virus [32]. Likewise, SARS-CoV-2 RNA was not detected in 51 NHPs sampled before and during the pandemic in Minas Gerais and Rio Grande do Sul [12]. Experimental infection studies have shown that *Callithrix jacchus*, a species found in Brazil and phylogenetically closely related to *Callithrix penicillata*—the primary species analyzed in our study—exhibits resistance to SARS-CoV-2. This resistance is characterized by mild symptoms and rapid viral RNA clearance [33,34], which may partially explain the absence of positive results in our analyses.

In contrast, infections have been reported in captive NHPs, where frequent, prolonged, and close contact with human caretakers appears to significantly increase the likelihood of infection [8,23,35,36]. Additionally, infection dynamics may vary among NHP species. For example, severe and fatal SARS-CoV-2 infections were recently observed in *Lagothrix lagothricha*, a critically endangered species [22]. This case highlights the complexity of primate responses to SARS-CoV-2 and underscores the need for continued surveillance, particularly in Brazil, which harbors the world’s highest biodiversity of NHPs. Some of these species, such as *Alouatta guariba guariba*, are critically endangered [37].

Small synanthropic mammals (SSMs) play a role in the transmission cycles of various zoonotic pathogens and, since the onset of the COVID-19 pandemic, have been considered potential reservoirs of SARS-CoV-2 [15]. Evidence of virus transmission from hamsters to humans [38] has further raised concerns about their role in the dissemination of this pathogen.

In the present study, we analyzed 18 individuals of *Rattus rattus* and six of *Rattus norvegicus*, two species previously identified as potential reservoirs of SARS-CoV-2. In Ecuador, an infection rate of 5% has been reported in these rodents, underscoring their epidemiological significance [10]. In Mexico, the presence of the virus has been detected in urban rodents, with infection rates of 12.1% in *Mus musculus* and 5.8% in *R. norvegicus* collected from water channels [39].

These findings are not limited to rodents. Another notable example within South America’s synanthropic fauna is the coati (*Nasua nasua*), which has also shown susceptibility to SARS-CoV-2. A study conducted in Minas Gerais, Brazil, detected natural infections in 5% of coatis through the analysis of anal swabs, highlighting the importance of these animals in SARS-CoV-2 epidemiological surveillance [31].

To date, no studies have reported the presence of SARS-CoV-2 in *Didelphis albiventris* (white-eared opossum), another small synanthropic mammal commonly found in Brazil. However, Goldberg et al. (2024) [21] identified SARS-CoV-2 at the molecular level in *D. virginianus*, a closely related species. The study also revealed unique mutations in the receptor-binding domain (RBD) of the spike protein in *D. virginianus* samples, suggesting potential viral adaptations for transmission within this species. These findings underscore the need for a One Health approach to better understand the dynamics of SARS-CoV-2 transmission in wildlife. In Brazil, *D. albiventris* exhibits behavior similar to that of rodents and is frequently exposed to SARS-CoV-2 through indirect contact with humans, such as via contaminated sewage, food scraps, or household waste [24,25,26].

In our study, SSMs were collected from specific locations in the city of Diamantina, where SARS-CoV-2 RNA had been detected in sewage samples [29]. However, viral RNA was not detected in any of the tested animal samples, consistent with findings from Belgium, where *Rattus norvegicus* exposed to sewage containing SARS-CoV-2 also tested negative [40]. These results suggest that mere exposure does not necessarily lead to infection, highlighting the influence of other factors, such as genetic compatibility and local ecological conditions.

The absence of specific antibodies against SARS-CoV-2 in the analyzed samples indicates that these animals had not experienced significant exposure to the virus by the time of collection. This finding is crucial for understanding the dynamics of viral circulation in synanthropic populations, suggesting that no widespread prior infection had occurred in the studied locations. The lack of an immune response, associated with the absence of detection of viral RNA, supports the hypothesis that SARS-CoV-2 is not infecting those species. This is relevant not only for assessing potential zoonotic cycles but also for guiding epidemiological surveillance strategies and disease control efforts in the synanthropic fauna.

Diamantina, a small touristic municipality with approximately 45,000 inhabitants [41], has a sewage system that typically exhibits a relatively low SARS-CoV-2 viral load [29], which may explain the absence of infection in the sampled SSMs. However, future studies with larger sample sizes and broader geographic coverage are essential to provide a more comprehensive understanding of infection dynamics and exposure risks in these animals. Such investigations will generate more precise data to enhance disease surveillance and control strategies in synanthropic fauna.

Our study has some limitations that should be considered when interpreting the results. First, due to equipment and laboratory infrastructure constraints, we did not employ serological techniques such as PRNT for detecting SARS-CoV-2 in NHP samples, which could have provided evidence of past infections in these specimens [21,39]. Thus, it does not assess past exposure in NHPs but rather focuses on current infection status—a critical distinction. Additionally, we lacked access to fecal or anal swab samples from these animals, potentially limiting our findings, as viral RNA has been detected in such specimens [9,23,36]. Finally, sampling was concentrated in specific regions, including the Jequitinhonha Valley and Northern Minas Gerais, which may not be representative of other geographic areas with different epidemiological profiles.

Based on our data presented, there is no evidence that SARS-CoV-2 has established zoonotic cycles in the wild or synanthropic populations analyzed to date. This finding contributes to epidemiological surveillance and control efforts, particularly in high-biodiversity regions such as Brazil. However, given the virus’s high mutation rate and capacity to adapt to new hosts, continuous monitoring remains essential. Preventing potential zoonotic cycles requires sustained research efforts and public policies integrating human, animal, and environmental health. The integrated approach applied here serves as a model for future investigations and highlights the need to expand surveillance to additional regions, increase sample sizes, and incorporate serological and genomic techniques.

## Figures and Tables

**Table 1:** Species, samples, location, and date of sampling of mammals tested for SARS-CoV-2. Legend: O: oral/nasal swab; L: lung; BS: body swab; B: blood; Lv: liver; S: spleen; F: feces.

Mammal	Cod.	Species	Sample	Municipality	Sampling Point	Lat	Long	Environment	Sampling Date
NHP	097	*C. penicillata*	O	Salinas	random	−16.15637	−42.30730	Rural	17 March 2022
	098	*C. penicillata*	O	Salinas	random	−16.15637	−42.30730	Rural	17 March 2022
	099	*C. penicillata*	O	Salinas	random	−16.15637	−42.30730	Rural	17 March 2022
	100	*C. penicillata*	O	Salinas	random	−16.15637	−42.30730	Rural	22 March 2022
	101	*C. penicillata*	O	Salinas	random	−16.15637	−42.30730	Rural	22 March 2022
	102	*C. penicillata*	L	Francisco Dumont	random	−17.27788	−44.23403	Rural	30 March 2022
	103	*C. penicillata*	L	Bocaiuva	random	−17.07593	−43.93418	Rural	30 March 2022
	104	*C. penicillata*	L	Jequitaí	random	−16.98432	−44.35691	Rural	30 March 2022
	105	*C. penicillata*	L	São João da Lagoa	random	−16.82729	−44.32653	Sylvatic	30 March 2022
	106	*C. penicillata*	L	São João da Lagoa	random	−16.82729	−44.32653	Sylvatic	30 March 2022
	107	*C. penicillata*	O	Salinas	random	−16.15637	−42.30730	Rural	4 April 2022
	108	*C. penicillata*	O	Salinas	random	−16.15637	−42.30730	Rural	4 April 2022
	109	*C. penicillata*	O	Salinas	random	−16.15637	−42.30730	Rural	7 April 2022
	110	*C. penicillata*	O	Salinas	random	−16.15637	−42.30730	Rural	7 April 2022
	111	*C. penicillata*	O	Buenópolis	random	−17.87375	−44.18200	Urban	26 May 2022
	112	*C. penicillata*	O	Buenópolis	random	−17.87375	−44.18200	Urban	26 May 2022
	113	*C. penicillata*	O	Buenópolis	random	−17.87375	−44.18200	Urban	26 May 2022
	114	*C. penicillata*	L	Salinas	random	−16.15053	−42.30761	Rural	20 June 2022
	115	*C. penicillata*	O; L	Salinas	random	−16.15053	−42.30761	Rural	20 June 2022
	116	*C. penicillata*	L	Ubaí	random	−16.28660	−44.77993	Urban	29 June 2022
	117	*C. penicillata*	L	Ubaí	random	−16.28660	−44.77993	Urban	8 June 2022
	118	*C. penicillata*	L	Ubaí	random	−16.29680	−44.78357	Urban	17 June 2022
	119	*C. penicillata*	L	Ubaí	random	−16.29680	−44.78357	Urban	27 June 2022
	120	*C. penicillata*	L	Montes Claros	random	−16.65341	−43.89827	Sylvatic	6 March 2022
	121	*C. penicillata*	L	Guaraciama	random	−17.01723	−43.68094	Rural	27 April 2022
	122	*C. penicillata*	L	Olhos D’água	random	−17.39881	−43.56943	Urban	7 June 2022
	123	*C. penicillata*	L	Lagoa dos Patos	random	−17.01663	−44.78229	Rural	15 June 2022
	124	*C. penicillata*	L	Engenheiro Navarro	random	−17.28509	−43.95465	Urban	7 July 2022
	125	*C. penicillata*	L	Lassance	random	−17.88429	−44.57745	Urban	8 June 2022
	126	*C. penicillata*	L	Montes Claros	random	−16.74956	−43.89984	Rural	4 May 2022
	127	*C. penicillata*	L	Francisco Dumont	random	−17.50288	−44.12984	Rural	7 July 2022
	128	*C. penicillata*	L	Francisco Dumont	random	−17.31509	−44.23189	Urban	7 July 2022
	129	*C. penicillata*	L	Francisco Dumont	random	−17.50288	−44.12984	Rural	10 July 2022
	130	*C. penicillata*	L	Montes Claros	random	−16.73397	−43.87953	Urban	12 July 2022
	131	*C. penicillata*	L	Brasília de Minas	random	−16.21072	−44.43640	Urban	7 July 2022
	132	*C. penicillata*	L	Salinas	random	−16.15637	−42.30730	Rural	13 July 2022
	137	*C. penicillata*	L	Ubaí	random	−16.28485	−42.26081	Rural	11 September 2022
	138	*C. penicillata*	L	Ubaí	random	−16.28534	−44.78366	Rural	12 September 2022
	139	*C. penicillata*	L	Ubaí	random	−16.28504	−44.78340	Urban	13 September 2022
	140	*C. penicillata*	L	Ubaí	random	−16.28469	−44.78361	Urban	14 September 2022
	141	*C. penicillata*	L	Montes Claros	random	−16.72416	−43.83795	Urban	20 October 2022
	142	*C. penicillata*	L	Guaraciama	random	−17.01429	−43.66866	Urban	20 October 2022
	143	*C. penicillata*	L	Juramento	random	−16.84982	−43.58690	Urban	20 October 2022
	144	*C. penicillata*	L	São João da Lagoa	random	−16.85326	−44.34971	Urban	20 October 2022
	145	*C. penicillata*	L	São João da Lagoa	random	−16.85275	−44.35031	Urban	20 October 2022
	146	*C. penicillata*	L	São João da Lagoa	random	−16.85341	−44.34989	Urban	20 October 2022
	147	*C. penicillata*	L	São João da Lagoa	random	−16.85311	−44.35059	Urban	20 October 2022
	148	*C. penicillata*	L	Francisco Sá	random	−16.26377	−43.58839	Rural	20 October 2022
	149	*C. penicillata*	L	Francisco Sá	random	−16.26385	−43.58842	Rural	20 October 2022
	150	*C. penicillata*	L	São João da Lagoa	random	−16.85277	−44.35047	Urban	23 August 2022
	151	*C. penicillata*	L	São João da Lagoa	random	−16.85101	−44.34995	Urban	23 August 2022
	152	*C. penicillata*	L	São João do Pacuí	random	−16.53487	−44.53158	Urban	22 September 2022
	153	*C. penicillata*	L	São João do Pacuí	random	−16.53506	−44.53145	Urban	29 August 2022
	154	*C. penicillata*	L	São João do Pacuí	random	−16.53537	−44.53156	Urban	2 September 2022
	155	*C. penicillata*	L	São João da Lagoa	random	−16.85102	−44.34995	Urban	15 September 2022
	156	*C. penicillata*	L	Jequitaí	random	−17.23142	−44.44344	Urban	20 October 2022
	157	*C. penicillata*	L	Jequitaí	random	−17.23142	−44.44344	Urban	20 October 2022
	158	*C. penicillata*	L	São João da Lagoa	random	−16.85309	−44.35072	Urban	22 October 2022
	159	*C. penicillata*	L	Jequitaí	random	−17.23079	−44.44417	Urban	24 October 2022
	160	*C. penicillata*	L	Jequitaí	random	−17.23079	−44.44417	Urban	25 October 2022
	161	*A. caraya*	L	Salinas	random	−16.16133	−42.31082	Rural	26 October 2022
	162	*C. penicillata*	L	Salinas	random	−16.16287	−42.29934	Urban	14 October 2022
	166	*C. penicillata*	O	Bonito de Minas	random	−15.34867	−44.90012	Sylatic	10 February 2023
	167	*C. penicillata*	O	Bonito de Minas	random	−15.34867	−44.90012	Sylatic	10 February 2023
	168	*C. penicillata*	O	Bonito de Minas	random	−15.34867	−44.90012	Sylatic	10 February 2023
	169	*C. geoffroyi*	O	Teófilo Otoni	random	−17.89125	−41.52602	Rural	8 March 2023
	170	*C. geoffroyi*	O	Teófilo Otoni	random	−17.89125	−41.52602	Rural	8 March 2023
	171	*C. geoffroyi*	O	Teófilo Otoni	random	−17.89125	−41.52602	Rural	8 March 2023
	172	*C. geoffroyi*	O	Teófilo Otoni	random	−17.89125	−41.52602	Rural	8 March 2023
	173	*A. caraya*	O	Almenara	random	−16.18280	−40.69075	Urban	12 March 2023
	174	*A. caraya*	O	Almenara	random	−16.18280	−40.69075	Urban	12 March 2023
	175	*C. kuhlii*	O	Almenara	random	−16.15744	−40.69302	Urban	13 March 2023
	176	*C. kuhlii*	O	Almenara	random	−16.15744	−40.69302	Urban	13 March 2023
	177	*A. caraya*	O	Almenara	random	−16.15744	−40.69302	Urban	13 March 2023
	178	*C. kuhlii*	O	Almenara	random	−16.18280	−40.69075	Urban	14 March 2023
	179	*C. penicillata*	O	Salinas	random	−16.15475	−42.30760	Rural	23 February 2023
	180	*C. penicillata*	O	Salinas	random	−16.06398	−42.24160	Rural	18 May 2023
	182	*C. penicillata*	O	Salinas	random	−16.15475	−42.30760	Rural	20 June 2023
	183	*C. penicillata*	O	Salinas	random	−16.15475	−42.30760	Rural	13 July 2023
	185	*C. penicillata*	O	Salinas	random	−16.15475	−42.30760	Rural	25 July 2023
	186	*C. penicillata*	O	Araçuaí	random	−16.73491	−42.06367	Rural	20 September 2023
	187	*C. penicillata*	L	Salinas	random	−16.15475	−42.30760	Rural	5 October 2023
SSMs	D1	*D. albiventris*	O; BS; B; L; S; Lv; F	Diamantina	P1	−18.24400	−43.62300	Urban	25 June 2021
	D2	*R. rattus*	O; BS; B; L; S; Lv; F	Diamantina	P3	−18.25642	−43.60078	Urban	25 June 2021
	D3	*D. albiventris*	O; BS; B; L; S; Lv; F	Diamantina	P6	−18.25185	−43.58418	Urban	25 June 2021
	D4	*R. rattus*	O; BS; B; L; S; Lv; F	Diamantina	P4	−18.25900	−43.58435	Urban	25 June 2021
	D5	*R. rattus*	O; BS; B; L; S; Lv; F	Diamantina	P4	−18.25900	−43.58435	Urban	2 July 2021
	D6	*R. rattus*	O; BS; B; L; S; Lv; F	Diamantina	P4	−18.25900	−43.58435	Urban	2 July 2021
	D7	*D. albiventris*	O; BS; B; L; S; Lv; F	Diamantina	P5	−18.25650	−43.58195	Urban	2 July 2021
	D8	*R. rattus*	O; BS; B; L; S; Lv; F	Diamantina	P2	−18.24907	−43.61668	Urban	9 July 2021
	D9	*R. rattus*	O; BS; B; L; S; Lv; F	Diamantina	P4	−18.25900	−43.58435	Urban	9 July 2021
	D10	*R. rattus*	O; BS; B; L; S; Lv; F	Diamantina	P3	−18.25642	−43.60078	Urban	9 July 2021
	D11	*D. albiventris*	O; BS; B; L; S; Lv; F	Diamantina	P6	−18.25185	−43.58418	Urban	9 July 2021
	D12	*R. novergicus*	O; BS; B; L; S; Lv; F	Diamantina	P9	−18.22757	−43.61225	Urban	16 July 2021
	D13	*R. rattus*	O; BS; B; L; S; Lv; F	Diamantina	P2	−18.24907	−43.61668	Urban	16 July 2021
	D14	*R. rattus*	O; BS; B; L; S; Lv; F	Diamantina	P3	−18.25642	−43.60078	Urban	16 July 2021
	D15	*D. albiventris*	O; BS; B; L; S; Lv; F	Diamantina	P3	−18.25642	−43.60078	Urban	16 July 2021
	D16	*R. rattus*	O; BS; B; L; S; Lv; F	Diamantina	P4	−18.25900	−43.58435	Urban	16 July 2021
	D17	*R. rattus*	O; BS; B; L; S; Lv; F	Diamantina	P6	−18.25185	−43.58418	Urban	16 July 2021
	D18	*D. albiventris*	O; BS; B; L; S; Lv; F	Diamantina	P7	−18.24375	−43.59173	Urban	16 July 2021
	D19	*D. albiventris*	O; BS; B; L; S; Lv; F	Diamantina	P3	−18.25642	−43.60078	Urban	23 July 2021
	D20	*D. albiventris*	O; BS; B; L; S; Lv; F	Diamantina	P5	−18.25650	−43.58195	Urban	29 July 2021
	D21	*D. albiventris*	O; BS; B; L; S; Lv; F	Diamantina	P5	−18.25650	−43.58195	Urban	29 July 2021
	D22	*R. rattus*	O; BS; B; L; S; Lv; F	Diamantina	P6	−18.25185	−43.58418	Urban	29 July 2021
	D23	*D. albiventris*	O; BS; B; L; S; Lv; F	Diamantina	P5	−18.25650	−43.58195	Urban	30 July 2021
	D24	*D. albiventris*	O; BS; B; L; S; Lv; F	Diamantina	P7	−18.24375	−43.59173	Urban	6 August 2021
	D25	*R. rattus*	O; AS; B; L; S; Lv; F	Diamantina	P8	−18.23735	−43.59505	Urban	6 August 2021
	D26	*R. rattus*	O; BS; B; L; S; Lv; F	Diamantina	P8	−18.23735	−43.59505	Urban	27 August 2021
	D27	*R. rattus*	O; BS; B; L; S; Lv; F	Diamantina	P2	−18.24907	−43.61668	Urban	2 September 2021
	D28	*M. musculus*	O; BS; B; L; S; Lv; F	Diamantina	P2	−18.24907	−43.61668	Urban	2 September 2021
	D29	*R. rattus*	O; BS; B; L; S; Lv; F	Diamantina	P3	−18.25642	−43.60078	Urban	2 September 2021
	D30	*R. rattus*	O; BS; B; L; S; Lv; F	Diamantina	P4	−18.25900	−43.58435	Urban	2 September 2021
	D31	*D. albiventris*	O; BS; B; L; S; Lv; F	Diamantina	P5	−18.25650	−43.58195	Urban	2 September 2021
	D32	*D. albiventris*	O; BS; B; L; S; Lv; F	Diamantina	P5	−18.25650	−43.58195	Urban	2 September 2021
	D33	*R. rattus*	O; BS; B; L; S; Lv; F	Diamantina	P8	−18.23735	−43.59505	Urban	3 September 2021
	D34	*R. novergicus*	O; BS; B; L; S; Lv; F	Diamantina	P9	−18.22757	−43.61225	Urban	15 September 2021
	D35	*R. novergicus*	O; BS; B; L; S; Lv; F	Diamantina	P9	−18.22757	−43.61225	Urban	15 September 2021
	D36	*R. novergicus*	O; BS; B; L; S; Lv; F	Diamantina	P10	−18.22757	−43.61225	Urban	16 September 2021
	D37	*R. novergicus*	O; BS; B; L; S; Lv; F	Diamantina	P10	−18.24907	−43.61668	Urban	24 September 2021

## Data Availability

The original contributions presented in this study are included in the article. Further inquiries can be directed to the corresponding authors.
